# Correct approach in urticarial vasculitis made early diagnosis of lupus nephritis possible: a case report

**DOI:** 10.1186/s13256-022-03477-6

**Published:** 2022-08-22

**Authors:** Kyra Smets, Anne Van Baelen, Ben Sprangers, Petra De Haes

**Affiliations:** 1 Department of Dermatology, University Hospitals Leuven–KU Leuven, Herestraat 49, 3000 Leuven, Belgium; 2 Department of Nephrology, University Hospitals Leuven, Leuven, Belgium

**Keywords:** Systemic lupus erythematosus, Hypocomplementemia, Urticarial vasculitis, Lupus nephritis, Case report

## Abstract

**Background:**

Urticarial vasculitis is a clinicopathologic entity defined by recurrent episodes of urticarial lesions that persist > 24 hours and demonstrate the histopathologic features of leukocytoclastic vasculitis. The most important prognostic feature is the presence of normo- or hypocomplementemia. In the latter, patients are much more likely to have systemic manifestations. Urticarial vasculitis is most often idiopathic, but it can arise in association with autoimmune connective diseases, cryoglobulinemia, infections, medications, and hematologic malignancies.

**Case presentation:**

We present the case of a 61-year-old Caucasian woman with a skin eruption that consisted of erythematous plaques on the trunk and limbs that lasted > 24 hours but were asymptomatic. The skin eruption had an acute onset and persisted for 3 months upon initial presentation in our dermatology department. A punch biopsy showed signs of a leukocytoclastic vasculitis in the superficial dermis. On laboratory examination, signs of activation of the complement system were found with low complement C3, C4, and C1q, and with a high anti-C1q antibody titer. The clinical, histological, and lab results fit the diagnosis of hypocomplementemic urticarial vasculitis. There was also a positive antinuclear factor with elevated U1 small nuclear ribonucleoprotein and high double-stranded DNA determined by Farr method. On urinalysis, marked proteinuria and massive hematuria were found. Kidney biopsy showed focal crescentic and focal mesangial type of glomerular damage with a full-blown positivity of immunoglobulin A, immunoglobulin G, and C1q, leading to lupus nephritis class III-A (according to the International Society of Nephrology/Renal Pathology Society 2003 classification of lupus nephritis). The patient was treated with hydroxychloroquine, corticosteroids, and low-dose intravenous cyclophosphamide (Euro-Lupus regimen) as remission-inducing agent, followed by azathioprine as remission-maintaining agent. This treatment regimen gave good results, with total clearance of the skin lesions and remission of the lupus nephritis.

**Conclusion:**

Clinicopathologic recognition of urticarial vasculitis with correct screening for extracutaneous disease can lead to early diagnosis of serious organ involvement and thereby improve prognosis for the patient.

## Background

Urticarial vasculitis is a variant of cutaneous small-vessel vasculitis. The cutaneous features consist of erythematous plaques or wheals that clinically resemble urticaria, however, individual lesions persist for more than 24 hours. Lesions preferentially appear on the trunk and proximal extremities. In contrast to chronic urticaria, lesions are burning and painful rather than itchy, and often heal with post-inflammatory hyperpigmentation. The disease tends to occur in young to middle-aged women [1] with a peak incidence in the fourth decade of life [2].

The most important prognostic feature in urticarial vasculitis is the presence of normo- or hypocomplementemia. In the latter, patients are much more likely to have systemic manifestations. Hypocomplementemic urticarial vasculitis syndrome (HUVS) is a more severe syndrome with extensive organ damage [2].

Extracutaneous symptoms can be musculoskeletal, pulmonary, renal, or gastrointestinal. Among the most common are musculoskeletal manifestations (arthralgia, arthritis) with pulmonary involvement being the leading cause of morbidity and mortality in these patients [1].

Urticarial vasculitis is most often idiopathic, but it can arise in association with autoimmune connective tissue diseases [especially Sjogren’s syndrome and systemic lupus erythematosus (SLE)], serum sickness, cryoglobulinemia, infections, medication, and hematologic malignancies. Hypocomplementemic urticarial vasculitis (HUV) is often associated with SLE (54%), but normocomplementemic urticarial vasculitis (NUV) is not (2%) [3]. HUVS might resemble SLE, but it can be differentiated by the presence of ocular inflammation (episcleritis, conjunctivitis), angioedema, and COPD-like symptoms [2].

We present the case of a middle-aged woman in whom the diagnosis of urticarial vasculitis was made, and owing to comprehensive screening, lupus nephritis was discovered.

## Case presentation

### History

A 61-year-old Caucasian woman was referred to our dermatology department in July 2019 because of a skin eruption that had initiated 3 months earlier. In April there was an acute onset of erythematous macules and papules on both thighs, which later spread to the back, arms, and the back of the hands. Treatment with topical steroids of medium potency (mometasone furoate 0.1%) and hydrating creams was inefficient.

Individual lesions tended to last for approximately 2 days before regression, but were asymptomatic. There were no bullae or erosions, nor mucosal lesions. Exposure to the sun aggravated the skin eruption.

The patient did not experience musculoskeletal, gastrointestinal, pulmonary, or any other symptoms. There was no family history of autoimmune or other relevant diseases.

### Physical findings

**Fig. 1:**
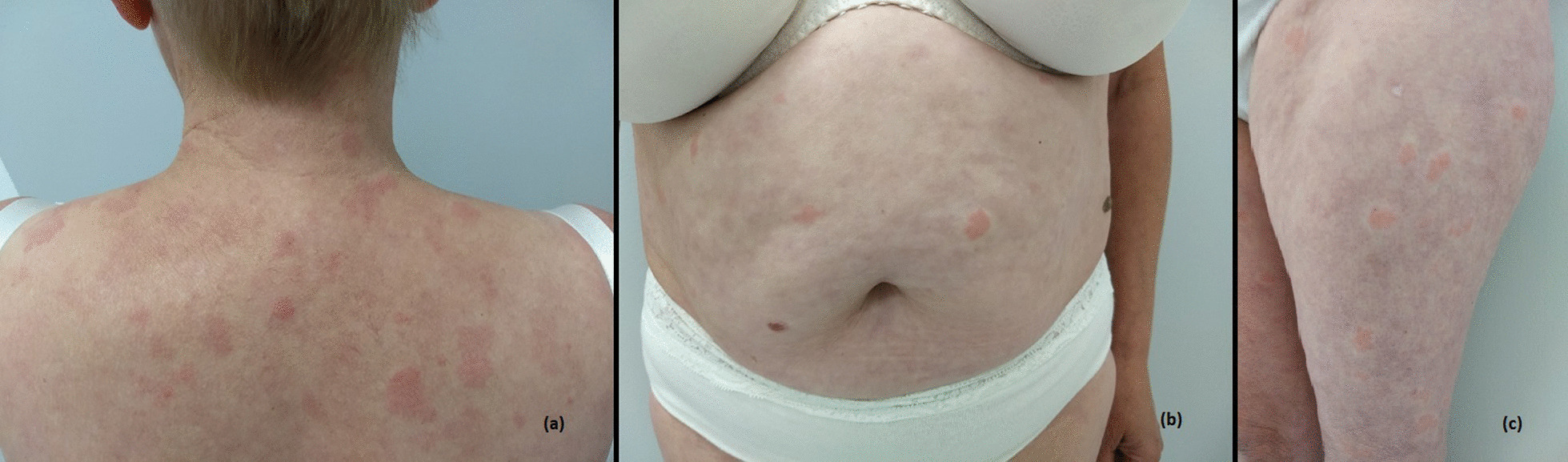
Patient presenting with a skin eruption with acute onset consisting of multiple sharply demarcated erythematous plaques on the back (**a**), chest, abdomen (**b**), and legs (**c**), and somewhat less on the arms and hands. There were no lesions on the groin, palmoplantar, or on the scalp.

On clinical examination, we found multiple sharply demarcated erythematous plaques on the back, chest, abdomen, and legs, and somewhat less on the arms and hands. There were no lesions on the groin, palmoplantar, or on the scalp (Fig. 1a–c).

### Skin biopsy

A 4-mm punch biopsy had already been taken by the referring dermatologist and showed no epidermal abnormalities. In the superficial dermis, however, there was a perivascular inflammatory infiltrate consisting of neutrophils, some histiocytes, lymphocytes, and eosinophils. There was remarkable leukocytoclasis. The vascular endothelium was mildly swollen but without any fibrinoid necrosis of the vascular walls.

### Laboratory data

Complete blood count showed white blood cells of 10.80 × 10^9^/L with an increase in neutrophils (8.7 × 10^9^/L), but a normal red blood cell and platelet count. The erythrocyte sedimentation rate (ESR) was 31 mm and C-reactive protein 7.6 mg/L. The alanine aminotransferase (ALAT) and gamma glutamyl transferase (gammaGT) were mildly increased (ALT 33 U/L, gammaGT 41 U/L). Antinuclear factor (ANF) was positive (1:320) with dsDNA Farr titer of 38.9 IU/mL (normal range < 7.0 IU/mL). Lupus anticoagulant, anticardiolipine immunoglobulin M (IgM), anti-Streptolysine O antibodies, and hepatitis B and C serology were negative. Antineutrophil cytoplasmic antibodies (c-ANCA) was negative, perinuclear antineutrophil cytoplasmic antibodies (p-ANCA) was 1/160 but with negative identification of proteinase 3 and myeloperoxidase. Cryoglobulins were present but without monoclonal fractions. Complement analysis showed hypocomplementemia with low component C3 fraction (C3) of 0.58 g/dL (normal range, 0.79–1.52 g/dL) and low component C4 fraction (C4) of 0.06 g/dL (normal range, 0.16–0.38 g/dL). The titer of C1q was 0.09 g/dL (normal range, 0.10–0.26 g/dL) and the anti-C1q antibodies > 400 U/mL (normal range < 15 U/mL). Urinalysis showed proteinuria 2+ and massive hematuria, but also a white blood cell count of 32/µL (normal range ≤ 10).

### Diagnosis and treatment

On the basis of the clinical image, histopathology, and lab results, a diagnosis of hypocomplementemic urticarial vasculitis was made. Additional chest radiography and spirometry were normal. Because of the positive ANF with high dsDNA Farr titer, and proteinuria with massive hematuria on urinalysis, an underlying systemic lupus erythematosus (SLE) was suspected. A treatment consisting of high potency topical steroids (betamethasone dipropionate 0.05%) and systemic hydroxychloroquine (200 mg twice daily) was initiated, and the patient was referred to the nephrology department for further evaluation of the kidney function.

### Renal evaluation

More comprehensive laboratory investigations showed a serum creatinine of 0.82 mg/dL and calculated glomerular filtration rate of 77 mL/minute/1.73 m^2^ (CKD-EPI), which corresponds with chronic renal disease GFR category G2 (KDIGO guidelines). Twenty-four-hour urinalysis showed proteinuria of 0.33 g. Ultrasound showed normal kidney size. Because of the pathological proteinuria, along with massive hematuria and increased white blood cell count, hypocomplementemia and positive ANF (U1RNP positive), a kidney biopsy was performed.

Histopathological evaluation showed focal crescentic and focal mesangial type of glomerular damage. Immunohistochemically there was a full-blown positivity of immunoglobulin A (IgA), immunoglobulin G (IgG), and C1q. These features fit the diagnosis of lupus nephritis class III-A [according to the International Society of Nephrology and the Renal Pathology Society (ISN/RPS) 2003 classification of lupus nephritis].

Therapy according to the Euro-Lupus protocol was initiated, consisting of an intravenous steroid pulse (500 mg/day for 3 days), followed by oral methylprednisolone 0.8 mg/kg/day during the first month, afterwards slowly tapering the dose to 0.1 to 0.2 mg/kg/day. Cyclophosphamide (500 mg/day) was given six times, with an interval of 2 weeks. Along with this therapy, protective measures for gastrointestinal disease, candida infections, *Pneumocystis jirovecii* pneumonia, herpes infection, and osteoporosis was started.

With this treatment regimen, disease control was obtained with complete clearance of the skin eruption and a normal urinalysis without proteinuria nor hematuria within 3 months after the start of treatment. DNA Farr titer became negative, and there was no more complement consumption. Steroids were tapered, and azathioprine as maintenance therapy was initiated at a dose of 1 mg/kg/day with maintained disease control during 4 months of follow-up.

## Discussion and conclusion

Urticarial vasculitis is a small-vessel vasculitis, classified as an immune complex-mediated or type III hypersensitivity reaction [4]. It was first reported by McDuffie *et al*. in 1973, who described a syndrome in which urticaria was the single predominant clinical manifestation of leukocytoclastic vasculitis [5]. Histologically, according to Davis *et al.*, there should be evidence of leukocytoclasis and vessel wall destruction, which may or may not be accompanied by fibrinoid deposits. Red blood cell extravasation and perivascular inflammatory cell infiltrate may also be present [2].

We present the case of a 61-year-old Caucasian woman with hypocomplementemic urticarial vasculitis leading to the diagnosis of lupus nephritis owing to comprehensive screening for organ damage. The case therefore fulfilled the two major (urticarial vasculitis skin lesions, hypocomplementemia in serum) and three minor (venulitis of the dermis, glomerulonephritis, and a positive C1q precipitin test) criteria of McDuffie *et al*. [5, 6] (Table 1). However, the presence of significant cryoglobulinemia, an elevated anti-DNA antibody titer, and a high titer of ANF excludes the diagnosis of hypocomplementemic urticarial vasculitis syndrome [5]. In this case, the hypocomplementemic urticarial vasculitis was the presenting symptom of systemic lupus erythematosus.

**Table 1:** Diagnostic criteria of hypocomplementemic urticarial vasculitis syndrome from McDuffie *et al*. for the diagnosis of HUVS, two major criteria and at least two minor criteria should be present [6]

Major criteria	1. Urticarial vasculitic skin lesions 2. Low levels of serum complement (all components)
Minor criteria	1. Venulitis of the dermis on biopsy 2. Arthritis or arthralgia 3. Glomerulonephritis 4. Episcleritis or uveitis 5. Recurrent abdominal pain 6. A positive C1q precipitin test by immunodiffusion with an associated suppressed C1q level

Exclusion criteria are significant cryoglobulinemia (cryocrit > 1%), an elevated anti-DNA antibody titer, a high titer of antinuclear antibody (ANA), hepatitis B antigenemia, and hereditary deficiency of a complement component or of C1 esterase inhibitor

### Role of C1q and anti-C1q antibodies

As mentioned above, the most important prognostic feature and predictor of systemic involvement in urticarial vasculitis is the presence of normo- or hypocomplementemia. In addition, anti-C1q antibodies are present in HUV. These are antibodies that bind to the collagen-like region of C1q, leading to lowered C1q levels [7]. C1q functions as the initial step of the classical complement pathway activation and is essential for the removal of apoptotic cells and cellular debris [8]. Autoantibodies to C1q are not only associated with HUV(S), but also with SLE. Less frequently, these antibodies can also be found in other immune complex-mediated diseases, such as Felty’s syndrome, rheumatoid vasculitis, Sjögren’s syndrome, membranoproliferative glomerulonephritis, and IgA nephropathy [8].

Anti-C1q antibodies also have a prevalence of 3–5% in normal individuals [8]. However, they are detected at a high titer in 100% of patients with HUV and in 30–48% of SLE patients [8]. In SLE, their titer correlates with active renal disease with a sensitivity of 44–100% and a specificity of 70–92% [8]. It has therefore been suggested that an increase in anti-C1q antibody titer can predict renal flares in lupus nephritis, and that monitoring anti-C1q might be a valuable noninvasive biological marker in SLE patients [7, 8].

Autoantibodies to the collagen-like region of C1q have also been shown to deposit and concentrate in the glomeruli of patients with SLE and proliferative glomerulonephritis, potentially contributing to the pathogenesis of the renal disease [11, 12].

### Association between HUVS and SLE

HUVS is present in 7–8% of SLE patients and 54% of HUVS patients are diagnosed with SLE in the follow-up period [3]. Many features of HUVS overlap with SLE, and according to Davis *et al*., 54% of HUVS patients are later diagnosed with SLE in the follow-up period. Therefore, these authors suggest that HUVS is a subtype of SLE. Nevertheless, the findings of malar rash, photosensitivity, oral ulcers, lung fibrosis, and detection of antibodies to double-stranded DNA or leuko- and thrombopenia suggest SLE [13]. On the other hand, symptoms of angioedema, chronic obstructive pulmonary disease (COPD), and ocular inflammation (especially of the uveal tract) are characteristic of HUVS [2]. Although up to a third of patients with SLE have circulating anti-C1q antibodies and up to half of the patients with HUVS have a positive ANF, patients with HUVS do not have anti-dsDNA or anti-Sm antibodies [2, 14].

### Diagnosis and treatment

In the approach for diagnosis of a possible case of urticarial vasculitis, the first step is a biopsy of an early lesion (< 24 hours) for histologic examination. After establishing the diagnosis, minimal laboratory investigations should be exerted: complete blood count, erythrocyte sedimentation rate, urea, electrolytes, serum creatinine, urinalysis, liver function tests, serum complement levels (C1q, C3, C4), cryoglobulins, and hepatitis B and hepatits C serology [15]. Further screening for systemic disease should be done on the basis of the patient’s medical history or laboratory findings, especially in hypocomplementemic forms.

Treatment of urticarial vasculitis is often difficult and based on the severity of disease. In patients with sole cutaneous disease, antihistamine therapy can be used for symptomatic control of pruritus. If insufficient, indomethacin, colchicine, dapsone, and hydroxychloroquine can be added. When intermittent exacerbations of (extra-)cutaneous disease are present, patients sometimes require a course of corticosteroids. In patients with severe corticosteroid resistant urticarial vasculitis or with flagrant corticosteroid morbidity, other immunosuppressive agents such as azathioprine, cyclophosphamide, or cyclosporine may be required [11].

In conclusion, our patient presented with urticarial vasculitis as the presenting symptom of underlying SLE. The exact association between urticarial vasculitis and SLE, both immune complex-mediated disorders, remains to be further elucidated. Nevertheless, correct screening for systemic manifestations and possible SLE is mandatory in all patients with hypocomplementemic urticarial vasculitis.

## Data Availability

Data sharing is not applicable to this article as no datasets were generated or analyzed during the current study.

## Abbreviations

HUV: Hypocomplementemic urticarial vasculitis
HUVS: Hypocomplementemic urticarial vasculitis syndrome
SLE: Systemic lupus erythematosus
